# Influence of Estimated Training Status on Anti and Pro-Oxidant Activity, Nitrite Concentration, and Blood Pressure in Middle-Aged and Older Women

**DOI:** 10.3389/fphys.2017.00122

**Published:** 2017-03-07

**Authors:** André M. Jacomini, Danielle da Silva Dias, Janaina de Oliveira Brito, Roberta F. da Silva, Henrique L. Monteiro, Susana Llesuy, Kátia De Angelis, Sandra L. Amaral, Anderson S. Zago

**Affiliations:** ^1^Department of Physical Education, School of Science, São Paulo State UniversityBauru, Brazil; ^2^Translational Physiology Laboratory, Universidade Nove de JulhoSão Paulo, Brazil; ^3^Department of General and Inorganic Chemistry, University of Buenos AiresBuenos Aires, Argentina

**Keywords:** oxidative stress, blood pressure, exercise, functional fitness, aging

## Abstract

The purpose of this study was to compare the association between anti and pro-oxidant activity, nitrite concentration, and blood pressure (BP) in middle-aged and older women with different levels of estimated training status (TS). The sample consisted of 155 females (50–84 years) who were submitted to a physical examination to evaluate estimated TS through the “Functional Fitness Battery Test,” BP measurements, and plasma blood samples to evaluate pro-oxidant and antioxidant activity and nitrite concentrations. Participants were separated by age into a middle-aged group (<65 years) and an older (≥65 years) group and then subdivided in each group according to TS. Blood biochemistry was similar between groups. On the other hand, protein oxidation was lower in participants with higher TS, independent of age. Older females with higher TS presented higher nitrite concentrations, lower lipoperoxidation, and lower values of BP compared with those with lower TS. Lower GPx activity was observed in participants with higher TS compared with middle-aged with lower TS. Thus, our results suggest that good levels of TS may be associated with lower oxidative stress and higher nitrite concentration and may contribute to maintain normal or reduced blood pressure values.

## Introduction

Aging is a normal process that can be characterized as an association of many deleterious alterations in molecules, cells, and tissues, culminating in reduced efficacy of physiological functions, and loss of resistance or adaptability to stress (da Cruz et al., 2014). The Free Radical Theory of Aging proposed by Denham Harman (Harman, 1956) stipulates that high production of reactive oxygen species (ROS), such as superoxide anions (${\text{O}}_{2}^{-.}$), which can promote oxidative damage, culminating in cardiovascular injuries (Münzel et al., 2010; da Cruz et al., 2014; Wu et al., 2014; Mikhed et al., 2015). The balance between pro and antioxidant activity is well-established as an important physiological regulator of blood pressure (BP), and has an important role in the pathogenesis of hypertension (HT; Roberts and Sindhu, 2009; Virdis et al., 2011).

High antioxidant capacity provides appropriate defense against vascular oxidative stress, neutralizing free radicals and protecting nitric oxide (NO) from scavenging, thereby exerting beneficial effects on vascular function (Roberts and Sindhu, 2009; Mikhed et al., 2015) and consequently better BP control. However, higher oxidative stress has been reported during the aging process, probably caused by reduced expression or deficiency in the activity of endogenous antioxidants (Harman, 2003; ABESO, 2009; Del Pozo-Cruz et al., 2014) and the fast reaction between NO and ${\text{O}}_{2}^{-.}$ to generate peroxynitrite (ONOO^−^), which results in decreased NO bioavailability (Darley-Usmar et al., 1995; Kojda and Harrison, 1999; Steiner et al., 2002). Therefore, the interaction between ROS and NO compromises vascular homeostasis which is considered the major cause of impaired endothelium-dependent vasorelaxation in aging and in HT.

On the other hand, regular physical exercise has been considered a good strategy, not only to maintain general health, but also to decrease cardiovascular risks and BP levels due to its capacity to regulate anti and pro-oxidant activity and increase NO concentration (Darley-Usmar et al., 1995; Milanovic et al., 2013).

However, the overall effectiveness of physical exercise and its mechanisms to induce vascular health are still controversial. Gomes et al. (2008) did not find differences in plasma nitrite after 3 months of physical exercise in patients with metabolic syndrome and Bouzid et al. (2014) found differences in oxidative stress markers after acute exercise in a young group, but not in the older group. However, Santana et al. (2013) found a positive correlation between plasma nitrite concentration and physical exercise intensity. These controversial results, in part, could be due to the differences in type, intensity, frequency, and duration of the exercise program (Haskell et al., 2007; American College of Sports et al., 2009; Aidar et al., 2011), especially those related to older groups (AHA, 2001; Haskell et al., 2007; American College of Sports et al., 2009). Considering this and the current recommendations of the American College of Sports Medicine (ACSM) on the combination of different types of exercise for overall healthy aging (American College of Sports et al., 2009), it seems reasonable to suggest a multicomponent evaluation (endurance, flexibility, resistance, coordination, and others) to estimate training status (TS) instead of the evaluation of just one modality such as endurance or resistance training.

Although, these concepts are already individually established in the literature, there is limited evidence on the relationship between oxidative stress and BP control in middle-aged and older individuals with different levels of TS. With this background, the hypothesis of this study was that older individuals would present higher levels of pro-oxidant activity, which compromise NO concentration and BP, compared with the middle-aged group. However, when the individuals present high levels of TS, a lower level of BP would be observed due to better anti-oxidative capacity and NO concentration. Therefore, the purpose of this study was to compare the association between anti and pro-oxidant activity, NO concentration, and BP in middle-aged and older subjects with different levels of estimated TS.

## Methods

### Recruitment and screening

Researchers visited extension programs linked to universities and associations of the retired community and all individuals were invited to participate, with the same chance of being included in this study, if they met the inclusion criteria. Participants were required to be female, non-smoking, non-alcoholic, non-diabetic, aged between 50 and 85 years old, not have cardiovascular, peripheral, cerebrovascular, neurologic, or psychiatric diseases, present normal values of lipid and glycemic profile, and not have any other health conditions which could compromise performance in physical tests. Hypertensive participants were included in the study if blood pressure values were lower than 160 mmHg for systolic blood pressure (SBP) and 100 mmHg for diastolic blood pressure (DBP). All hypertensive participants were required to be under anti-hypertensive medicine treatment.

From all clusters visited, 155 subjects (67.14 ± 6.63 years) who met the inclusion criteria volunteered to participate in this study and were divided into 2 groups: middle-aged (<65 years old) and older individuals (≥65 years). The cut-off point to classify the participant as a middle-aged or older individual was 65 years, according to the international classification of the World Health Organization WHO (2015). The study was approved by the Institutional Review Board of São Paulo State University (CEP/FC-UNESP n° 323.427) and all subjects provided their written informed consent during the first laboratory visit, prior to beginning the experiments.

The sample size was calculated according to the prevalence of HT in the Brazilian population which is around 30% (SBH, 2010). The formula of Bolfarine and Bussab was used (Bolfarine and Bussab, 2005), which was performed with an 80% power and significance level of 5% (Singer, 1997; Hsieh et al., 1998).

### Plasmatic oxidative stress profile

Blood samples were collected by venous puncture in heparinized vacutainers after 12 h fasting, and immediately centrifuged at 4,000 rpm for 5 min for further analysis of plasma oxidant and antioxidant profile and nitrite concentration. During the blood collections care was taken to avoid hemolysis of blood. However, if hemolysis occurred, a new data collection was scheduled for the participant. All participants were advised to avoid foods rich in nitrates (beet, cabbage, spinach, and lettuce) throughout the day prior to blood collection.

### Protein assay

Protein concentration was determined according to the method previously described by Lowry et al. (1951), which uses bovine albumin solution at a concentration of 1 mg/mL as standard and 10 μl samples. This quantification was used to correct the calculation of the following analysis: endothelial superoxide dismutase (ecSOD), lipoperoxidation (LPO), and protein oxidation (Lowry et al., 1951).

### Lipoperoxidation (LPO)

Plasma lipid peroxide levels were determined by measuring thiobarbituric acid reactive substances (TBARS). This method is widely adopted to measure LPO through the concentration of malondialdehyde, the main breakdown product of oxidized lipids. For the TBARS assay, trichloroacetic acid (10%, w/v) was added to the homogenate to precipitate proteins and acidify the samples. This mixture was then centrifuged (4,000 rpm, 10 min), after which the protein-free sample was extracted and thiobarbituric acid (0.67%, w/v) added to the assay. The tubes were placed in a water bath (100°C) for 30 min, and the absorbance was measured at 535 nm using a spectrophotometer (Buege and Aust, 1978). Results are expressed as nmol/mg of protein.

### Protein oxidation

Protein oxidation was measured through carbonyls assay. Plasma samples were incubated with 2,4-dinitrophenylhydrazine (DNPH 10 mM) in a 2.5 M HCl solution for 1 h at room temperature in the dark, using 200 μl of the sample. Samples were mixed every 15 min. Next, 20% trichloroacetic acid (w/v) solution was added and the solution was incubated in ice for 10 min and centrifuged (2,000 rpm, 5 min). The supernatant was discarded and an additional wash with 10% trichloroacetic acid (w/v) was performed. The pellet was washed three times with ethanol ethyl acetate (1:1) (v/v). The final protein precipitates were dissolved in 6 M guanidine hydrochloride solution and incubated for 10 min at 37°C, and the absorbance was measured at 360 nm. Results are expressed as nmol/mg of protein (Reznick and Packer, 1994).

### Antioxidant enzyme activity

For the measurement of plasma ecSOD activity, a technique based on the inhibition of the reaction between superoxide radical and pyrogallol was used, a compound that oxidizes itself with pH variation, producing a colored product, detected spectrophotometrically at 420 nm. The inhibition percentage of initial reaction rates depends on the pH and quantity of ecSOD present in the reaction mixtures. Thus, the quantity of enzyme required to inhibit the reaction by 50% is defined as one unit of ecSOD. Three different concentrations of ecSOD (0.25, 0.5, and 1 U) were used to make the standard curve, which provided an equation of the line for purposes of calculation. Thus, ecSOD activity was determined by measuring the rate of formation of oxidized pyrogallol. Results are expressed as units of ecSOD per milligram of protein (U/mg protein; Fridovich, 1986).

The Glutathione peroxidase (GPx) activity was measured through an enzymatic-colorimetric method using a commercial kit (Cayman Chemical Company, Ann Arbor, MI, USA), by reaction with glutathione reductase, measuring the consumption of NADPH in the reduction reaction coupled to the GPx reaction. Results are expressed as nmol/min/mL.

### Nitrite concentration

Nitrites, metabolites of NO, were determined in plasma using Griess reagent in which a chromophore with a strong absorbance at 540 nm is formed by the reaction of nitrite with a mixture of naphthylethylenediamine (0.1%) and sulfanilamide (1%). A standard curve was established with a set of serial dilutions (10^−18^ to 10^−13^mol/l) of sodium nitrite as previously published (Granger et al., 1999). Results are expressed as nmol/L.

### Blood pressure (BP)

Blood pressure was measured after 5 min of rest on 3 separate days according to the VI Brazilian Hypertension Guidelines (SBH, 2010) and 2013 ESH/ESC Guidelines for the management of arterial hypertension (Mancia et al., 2013), using an aneroid sphygmomanometer adapted to the circumference of the arm and a stethoscope placed over the brachial artery.

### Physical examination

All subjects underwent a physical examination to assess body composition, anthropometric measurements, and estimated TS. The baseline blood test was performed at least 24 h after the final exercise test session.

#### Training status (TS)

To estimate the TS of the participants, the “Functional Fitness Battery Test” proposed by the “American Alliance for Health, Physical Education, Recreation and Dance” (AAHPERD) was performed. This battery test evaluates the following physical capacities: coordination, flexibility, muscular strength and endurance, dynamic agility, and cardiovascular endurance, as previously described (Zago and Gobbi, 2003; Benedetti et al., 2007; Mazo et al., 2010). Each test provides a percentile score, and the sum of these percentiles is used to calculate the general functional fitness index (GFFI) which is classified as very weak, weak, regular, good, or very good. However, due to low frequency of participants in the groups “very weak” and “very good,” and taking into account the minimum of participants required in each group to perform the statistical analysis, 10% (Hollander and Wolf, 1999), it was decided to include the participants classified as “very weak” and “weak” and the participants classified as “good” and “very good” in the same groups. Thus, all participants were divided according to age and TS into the following groups; TS1 (very weak and weak GFFI: score between 0 and 199), TS2 (regular GFFI: score between 200 and 299), and TS3 (good and very good GFFI: score between 300 and 500). The AAHPERD Battery Test demonstrates good reliability and criterion validity for use in older individuals. The test-retest reliability coefficients for each capacity have been reported in the range of *r* = 0.80–0.99 (Osness et al., 1990). Furthermore, this battery test was chosen because it includes multitask functional fitness which is in accordance with the recommendation of ACSM (American College of Sports et al., 2009) and presents good correlation between Vo_2_ max and functional fitness (*r* = 0.8/*p* < 0.01; Trapé et al., 2013).

#### Body composition

Body composition was measured through Dual energy x-ray absorptiometry scan (DEXA—Discovery Wi/HOLOGIC INC, Bedford, USA). After completion of the scans, the body composition was analyzed using the software settings, thus providing the results of total body fat mass.

#### Anthropometric indicators

Anthropometric measurements were analyzed through body weight and height using a stadiometer and electronic scale (Welmy-W200A-LED) and the body mass index (BMI) was calculated by dividing body weight (kg) by the square of the height (m).

### Statistical analysis

Results are presented as mean ± *SD*. One-way ANOVA was performed to evaluate statistical differences between the middle-aged (50–64 years) and older groups (65 years or more), with a significance level of *p* < 0.05. One-way multivariate analysis was performed considering the estimated TS (TS1, TS2, and TS3) as a group factor and age and BMI as covariate variables. The data were analyzed using the SPSS 20.0 statistical package.

## Results

Characteristics of the participants are shown in Table 1. The middle-aged group presented higher values of BMI compared with the older group. The middle-aged group also presented higher levels of TS measured by GFFI and lower values of SBP compared with the older group. No differences for blood biochemistry were found between the groups. Moreover, the percentage of older women not taking anti-hypertensive medication was lower compared with the middle-aged group, although the percentage of medication category was similar between groups. It is also important to note that medication which can influence pro and anti-oxidants (ACE inhibitor and AT1 receptors blockers) has the same percentage between groups, suggesting that it could exert the same effect in both group.

**Table 1:** **Characteristics of middle-aged and older participants**.

	**Middle-aged (*n* = 54)**	**Older (*n* = 101)**
Age (years)	60.44 ± 3.20	70.72 ± 5.01^*^
**ANTHROPOMETRY AND BODY COMPOSITION VARIABLES**		
Body mass index (kg/m^2^)	29.09 ± 4.65	27.39 ± 5.39^*^
Total body fat mass (%)	42.27 ± 5.26	38.67 ± 6.47
**FUNCTIONAL FITNESS**		
GFFI	260.62 ± 99.58	213.59 ± 106.57^*^
**BLOOD BIOCHEMISTRY**		
LPO (nmol/mg protein)	116.54 ± 76.88	119.06 ± 68.86
Proteinoxidation (nmol/mg protein)	2.73 ± 1.00	2.41 ± 0.96
ecSOD (U/mg protein)	1.10 ± 0.15	1.08 ± 0.15
GPx (nmol/min/mL)	0.4810 ± 0.16	0.4891 ± 0.16
Nitrite (nmol/L)	0.64 ± 0.49	0.54 ± 0.42
**BLOOD PRESSURE**		
SBP(mmHg)	119.60 ± 12.03	124.0 ± 12.94^*^
DBP (mmHg)	77.75 ± 7.62	76.02 ± 8.99
**ANTI-HYPERTENSIVE MEDICATION**		
Diuretics (%)	12.9	15.8
Adrenergic inhibitors (%)	3.7	12.8
Calcium channel blockers (%)	1.8	3.9
ACE inhibitors (%)	14.8	15.8
AT1 receptors blockers (%)	20.3	22.7
No medications (%)	46.5	29

*GFFI, general functional fitness index; LPO, lipoperoxidation; SBP, systolic blood pressure; DBP, diastolic blood pressure. Values are mean (SE)*.

* *p < 0.05*.

When each age group were divided according the estimated TS (TS1, TS2, and TS3), no difference was found in age, BMI and total body fat mass (Table 2). It only was observed differences for GFFI between TS groups.

**Table 2:** **Characteristics of middle-aged and older participants according estimated training status**.

	**Middle-aged (*n* = 54)**			**Older (*n* = 101)**		
	**TS1 (*n* = 18)**	**TS2 (*n* = 14)**	**TS3 (*n* = 22)**	**TS1 (*n* = 48)**	**TS2 (*n* = 28)**	**TS3 (*n* = 25)**
Age (years)	61.0 ± 2.4	61.3 ± 2.8	59.3 ± 3.7	72.0 ± 5.3	69.3 ± 4.4	69.8 ± 4.4
**ANTHROPOMETRY AND BODY COMPOSITION VARIABLES**						
Body mass index (kg/m^2^)	30.3 ± 4.6	28.4 ± 5.1	28.5 ± 4.3	28.5 ± 4.9	26.6 ± 7.2	25.9 ± 2.6
Total body fat mass (%)	43.8 ± 4.3	41.3 ± 5.8	41.2 ± 5.6	39.0 ± 7.2	39.6 ± 5.7	36.8 ± 5.3
**FUNCTIONAL FITNESS**						
GFFI	143.3 ± 29.3	255.3 ± 29.4^a^	359.9 ± 40.6^a^^b^	122.3 ± 47.6	238.6 ± 36.2^a^	360.6 ± 38.8^a^^b^

*GFFI, general functional fitness index; TS1, very weak and weak; TS2, regular; TS3, good and very good estimated training status. Values are mean (SE)*.

a p < 0.05 vs. TS1;

b *p < 0.05 vs. TS2*.

Due to the influence that age and BMI could exert in the results when participants were divided according to TS, both variables were considered as covariate variables in a multivariate analysis. However, no difference was found for age (*F* = 0.988/*p* > 0.05) and BMI (*F* = 1.027/*p* > 0.05) in middle-aged group and age (*F* = 0.896/*p* > 0.05) and BMI (*F* = 0.658/*p* > 0.05) for older group. MANOVA just showed effects in the TS groups (*F* = 1.820/*p* < 0.05 and *F* = 3.497/*p* < 0.001) for middle-aged and older groups, respectively.

**Figure 1:**
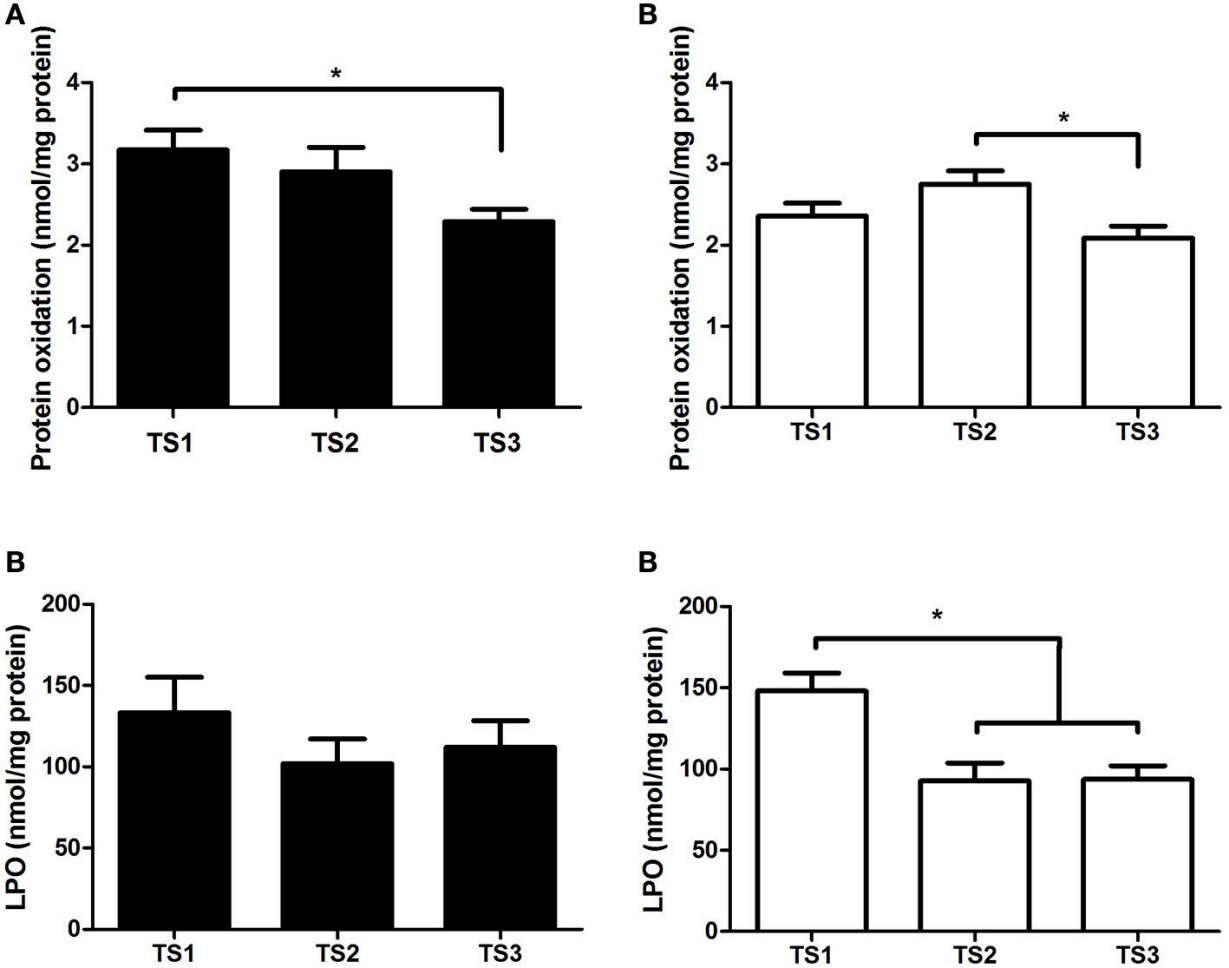
**Protein oxidation (A)** and lipoperoxidation (LPO) values **(B)** of middle-aged (black bars) and older individuals (white bars) with different levels of training status (TS1, very weak and weak; TS2, regular; TS3, good and very good). ^*^p < 0.05.

Bonferroni *post-hoc* test pointed out difference in oxidative profile variables (Figure 1). It can be observed that protein oxidation was lower in TS3 compared with TS1 in middle-aged and lower in TS3 compared with TS2 in older individuals. The LPO was lower in TS2 and TS3 compared with TS1 only for older individuals.

**Figure 2:**
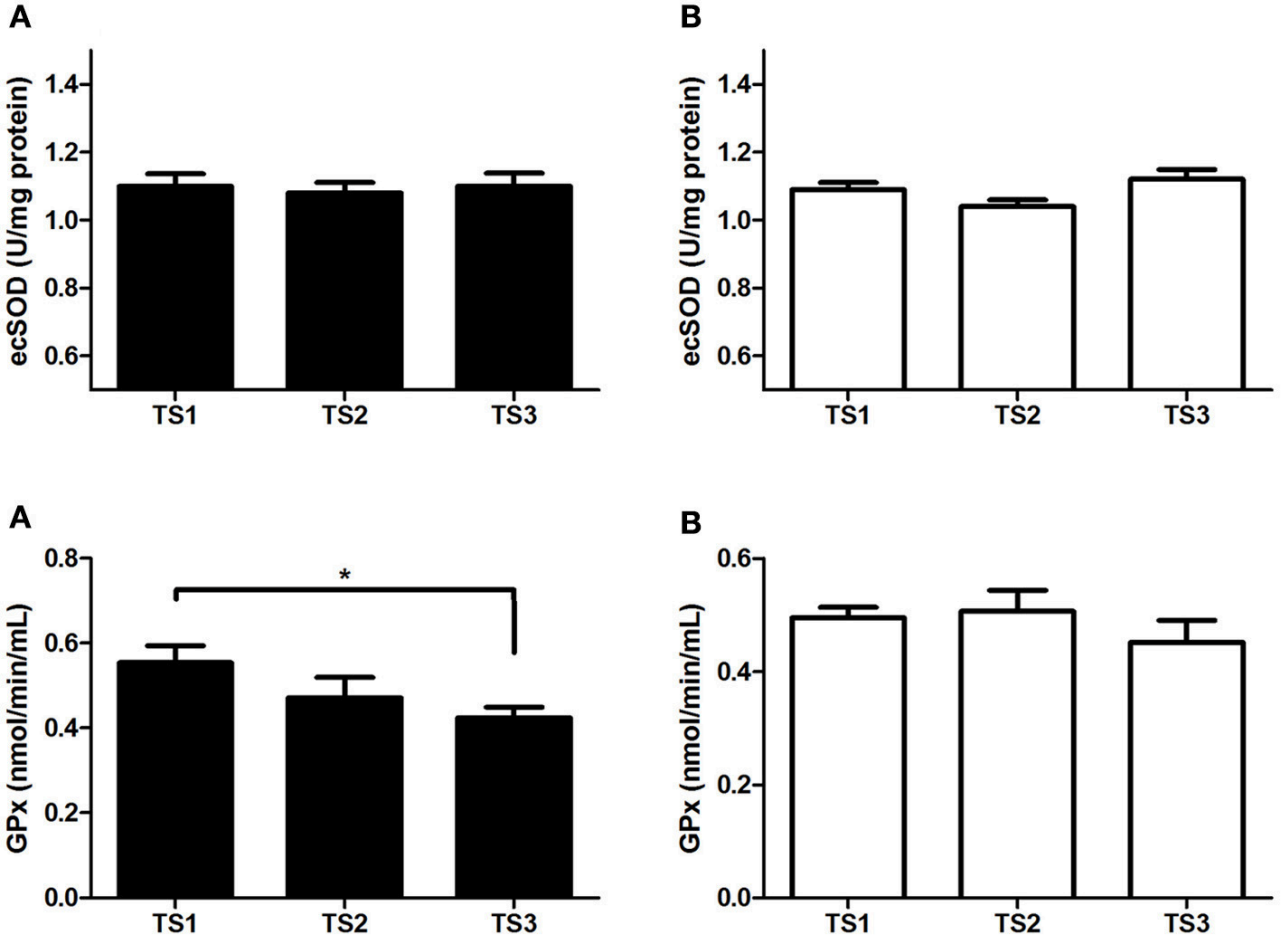
**ecSOD activity (upper panels) and GPx activity (bottom panels) of middle-aged** (**A**/black bars) and older individuals (**B**/white bars) with different levels of estimated training status (TS1, very weak and weak; TS2, regular; TS3, good and very good). ^*^p < 0.05.

In the antioxidant profile, no differences in ecSOD were found either in middle-aged or in older individuals. For GPx, the TS1 presented higher values compared with TS3 only in the middle-aged group (Figure 2).

**Figure 3:**
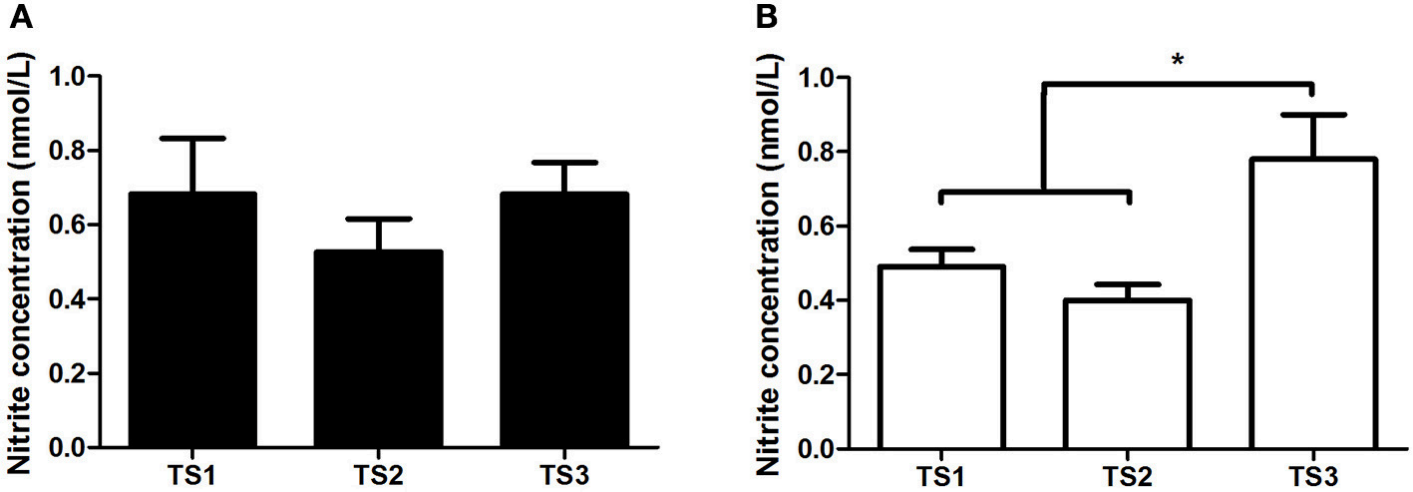
**Nitrite concentration of middle-aged** (**A**/black bars) and older individuals (**B**/white bars) with different levels of estimated training status (TS1, very weak and weak; TS2, regular; TS3, good and very good). ^*^p < 0.05.

**Figure 4:**
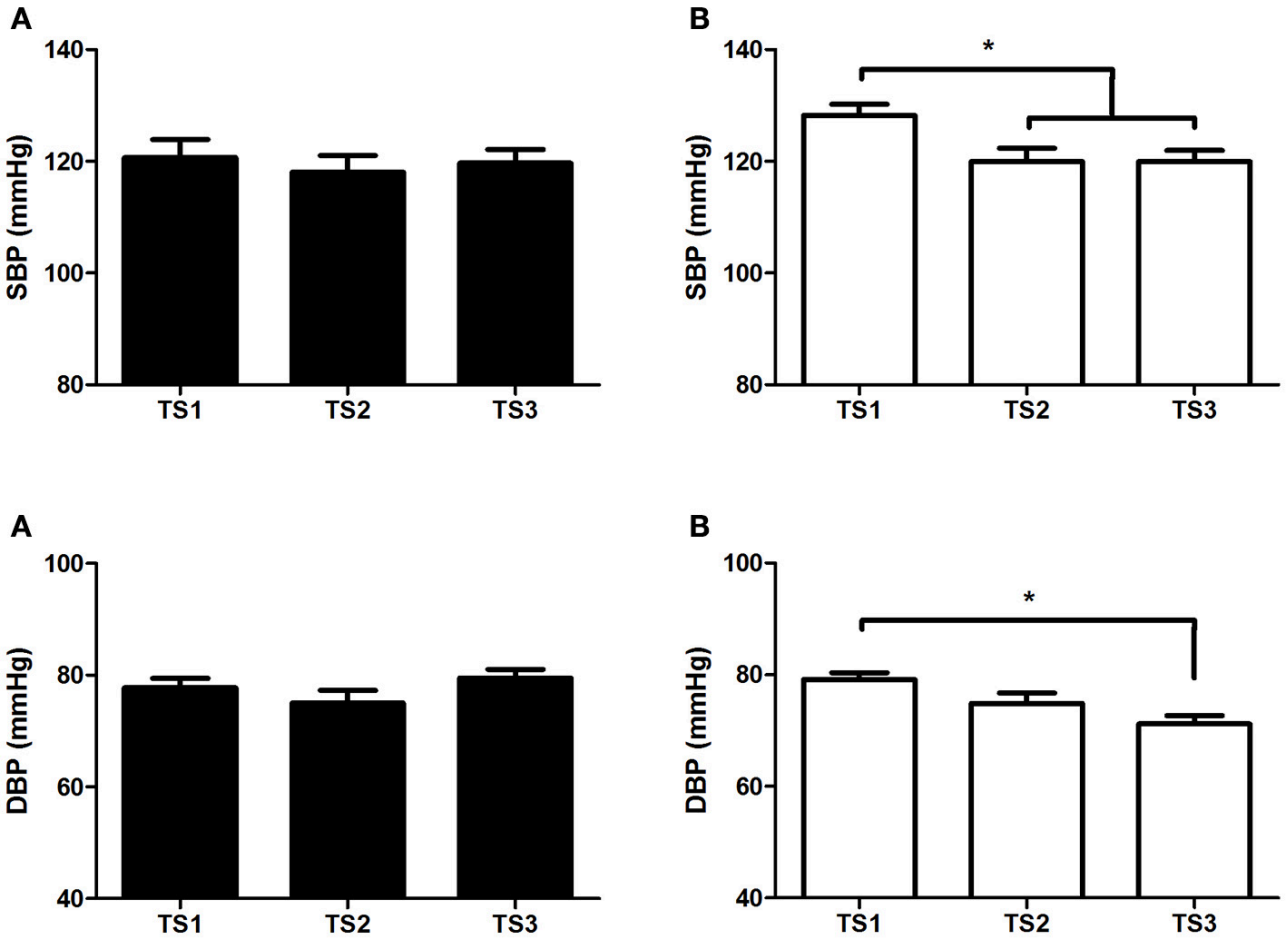
**Systolic blood pressure (upper panels) and diastolic blood pressure (bottom panels) of middle-aged** (**A**/black bars) and olderindividuals (**B**/white bars) with different levels of estimated training status (TS1, very weak and weak; TS2, regular; TS3, good and very good). ^*^p < 0.05.

In the middle-aged group, no differences were found in nitrite concentration even with different TS levels. However, TS3 showed higher plasma nitrite concentration compared with TS1 and TS2 in the older group (Figure 3). No differences were found for BP in the middle-aged group, while in the older group it can be observed that TS2 and TS3 presented lower values of SBP compared with TS1, and TS3 presented lower values of DBP compared with TS1 (Figure 4).

## Discussion

When comparing the association of oxidant variables between middle-aged and older individuals, the results presented in Table 1 do not demonstrate differences in blood biochemistry; however, the results of GFFI and SBP were better in the middle-aged group compared with the older group. Although, BMI values were higher in older individuals, both groups had the same classification according to the guidelines (WHO, 2000). Substantial published evidence shows that with the aging process, production of ROS significantly increases in the vasculature, which can promote an imbalance between the oxidant and antioxidant systems and cardiovascular injury (Wu et al., 2014). This is in agreement with the results of Okoduwa et al. in a study that compared hypertensive people of both genders, aged between 20 and 79 years, which showed that lower levels of endogenous antioxidant were directly related to aging (Okoduwa et al., 2015). Di Massimo et al. (2006) also found a significant negative correlation between ecSOD and age. However, contrary to these findings, Bouzid et al. (2014) did not find any difference in SOD or GPx activity between younger and older groups which agrees with the result to the current study.

The same behavior was found for nitrite concentration. The current study did not observe differences in this variable between middle-aged and older individuals, in accordance with the study of Lauer (Lauer et al., 2008), which showed no differences in plasma nitrite or nitrate, under baseline conditions, between middle-aged and older individuals. Nevertheless, Di Massimo et al. (2006) found a high negative correlation between nitrite/nitrate and age.

Regarding BP, the current study also did not find differences between middle-aged and older individuals, however it is important to note that in both groups the values were considered normal according to the guidelines (SBH, 2010; Mancia et al., 2013).

Despite studies in the literature reporting different results with respect to anti and pro-oxidant activity, nitrite concentration and the aging process, the level of TS was not evaluated in any of them. It is established that physical exercise is a good stimulus not only to increase the level of TS but also to promote better balance between anti and pro-oxidant activity and increase nitrite concentration (Jacomini et al., 2015). Therefore, with the purpose of comparing whether different levels of TS have enough power to promote alterations in these variables, the current study divided the middle-aged and older participants according to TS.

Analyzing the results of middle-aged separately, different levels of TS did not present any differences in LPO, ecSOD, nitrite concentrations, SBP, or DBP. Only protein oxidation and GPx presented lower levels in the TS3 compared with TS1. Differently, Bouzid et al. (2014) found no differences between young and older individuals in baseline ecSOD and GPx regardless the TS. However, these authors observed an increase in ecSOD and GPx results after an acute incremental exhaustive exercise performed on a cycle ergometer. In addition, González et al. (2008) showed that acute high intense aerobic exercise increases total antioxidant activity and decreases lipid hydroperoxide in healthy young individuals. It is important to note that these changes reported by Bouzid and González reflect a variation after acute exercise and not a chronic adaptation coming from higher levels of TS, however these results show the responsiveness of that variables front of acute exercise and current TS.

Although, ecSOD activity was not different between groups, GPx activity was higher in TS1 compared to TS3 in the middle-aged group. While ecSOD acts in ROS removal (Schneider and Oliveira, 2004; Rush et al., 2005), GPx catalyzes hydroperoxide (H_2_O_2_) reaction, avoiding ${\text{O}}_{2}^{\cdot -}$ and H_2_O_2_ accumulation. It contributes to low production of hydroxyl radicals and consequently low oxidative stress (Schneider and Oliveira, 2004; Weydert and Cullen, 2010; Araujo and Wilcox, 2014). However, the values of GPx in TS3 were lower when compared with TS1. The possible explanation for this is that GPx and catalase compete for the same substrate, being equally involved in H_2_O_2_ removal. Therefore, if the catalase has more reaction with H_2_O_2_, a lower level of GPx is expected. Thereby, higher activity of catalase could justify the result of GPx activity in the middle-aged group (Harman, 2003; ABESO, 2009; Conti et al., 2014; Del Pozo-Cruz et al., 2014). In the current study, catalase analysis was performed, but unfortunately the data were not presented due to high variability in the results, which can make the data unreliable.

A high level of protein oxidation was observed in TS1 compared with TS3 in the middle-aged group (Figure 1A), associated with higher GPx activity in TS1 (Figure 2), suggesting an up-regulation of this enzyme due to the higher necessity of H_2_O_2_ removal in TS1. This response could be interpreted as a positive feedback mechanism that reflects a response of exposure to oxidative stress in this group.

Overall, the results of the current study suggest that the TS is not a determinant factor for better values of pro and antioxidant profile, BP, or NO bioavailability in middle-aged. A possible explanation for this is the preserved endothelial function which provided fast adaptation against some stimulus or different levels of TS in the middle-aged.

On the other hand, in older subjects, it was observed that higher TS values represent lower exposure to oxidative stress and a high concentration of NO, although it did not represent better antioxidant activity, as shown in the present study. These results suggest that TS may acts as a protective effect against oxidative stress damage, which can contribute to higher NO bioavailability, probably due to lower NO scavenging and consequently better BP values. De Souza et al. using the method of intra-arterial infusions of acetylcholine and sodium nitroprusside, measured the responses on forearm blood flow by strain-gauge plethysmography. These authors observed that regular aerobic exercise can prevent the age-related loss in endothelium-dependent vasodilatation and restore levels in previously sedentary middle-aged and older healthy man (DeSouza et al., 2000). These results suggest that the age-associated loss in endothelium dependent vasodilatation can be prevented by regular aerobic exercise in both groups. It is in accordance with the current study which high level of TS were associated with higher NO concentration in the older group.

A regular level of TS (TS2) also decreased the exposure to oxidative stress (LPO) and SBP but no change was observed in nitrite concentration in older individuals. However, high levels of TS (TS3) not only decrease LPO and protein oxidation but also increase nitrite concentration and decrease SBP, DBP, and protein oxidation. Overall, TS is related to chronic adaptations to exercise and provides enough stimuli to modulate the balance between pro and antioxidant substances and increase NO concentration, which could be one of the important mechanisms that contribute to maintenance of good levels of SBP and DBP in the older TS3 group.

It is important to highlight that, even in individuals with blood pressure values considered normal, (due to anti-hypertension medicine or not) the older group demonstrated differences in blood pressure values between different TS groups. This result, *per se*, is really important as it points to a good level of TS as an important non-pharmacological tool against hypertension in older individuals.

Our results showed that TS was not enough to increase ecSOD values in either group (middle-aged and older individuals) or GPx values in the older group. Bouzid et al. found similar results after an incremental exhaustive exercise in middle-aged and older participants (Bouzid et al., 2014). These results suggest that antioxidant activity may have no sensitivity to respond to TS in older subjects. Besides, additional analysis of oxidative stress could provide greater support of the current results.

Non-pharmacological interventions for treatment and control of HT have emphasized the inclusion of physical exercises in daily routines (Hagberg et al., 2000). Moreover, this study suggests that regular physical exercise and, especially, the maintenance of high levels of TS are considered a protective effect against the deleterious effects of the aging process, thus ensuring greater autonomy and quality of life for older people (American College of Sports et al., 2009).

As limitations of the study, the authors point out that the methods used to determine LPO and protein oxidation are not the most current measures of systemic oxidative stress; nevertheless, these techniques are classical and widely clinically used by many researchers. We also acknowledge the potential influence of hemolysis on our measurements of SOD; however, it is unlikely that hemolysis influenced our results because we employed preventative precautions and rescheduled data collections if hemolysis occurred.

## Conclusions

This study suggests that maintenance of good levels of TS in the middle-aged group did not support differences in nitrite concentration or redox balance; although protein oxidation was lower in TS3. However, in older subjects, good levels of TS were associated with a lower response against oxidative stress arising from the aging process and higher nitrite concentration, which, in turn, could contribute to lower blood pressure values. These data suggest an important role of TS to avoid the interaction between ROS and NO, which may compromise vascular homeostasis and BP control.

## Ethics statement

Institutional Review Board of São Paulo State University. All subjects provided their written informed consent during their first laboratory visit, before the beginning of the experiments. This case does not apply to this study.

## Author contributions

AJ, DD, JB, and RD have made substantial contributions to acquisition, analysis, and interpretation of data. All of them have been involved in drafting the manuscript. HM, SL, KD, SA, and AZ have made substantial contributions to analysis and interpretation of data and have been involved in drafting the manuscript and revising it critically for important intellectual content. All authors read and approved the final manuscript.

### Conflict of interest statement

The authors declare that the research was conducted in the absence of any commercial or financial relationships that could be construed as a potential conflict of interest.
